# Complete genome of the multidrug-resistant *Acinetobacter baumannii* strain KBN10P02143 isolated from Korea

**DOI:** 10.1590/0074-02760160034

**Published:** 2016-05

**Authors:** Yong-Woon Lee, Hanna Choe, Sang-Heon Lee, Kyung Mo Kim, Sin Kam, Byung Kwon Kim, Won-Kil Lee

**Affiliations:** 1 Graduate School of Kyungpook National University, Department of Public Health, Daegu, Republic of Korea; 2 Korea Research Institute of Bioscience and Biotechnology, Microbial Resource Center, Jeongeup, Republic of Korea; 3 Kyungpook National University, School of Medicine, Department of Preventive Medicine, Daegu, Republic of Korea; 4 OmicsPia, Co. Ltd., Daejeon, Republic of Korea; 5 University of Science and Technology, Department of Bioinformatics, Daejeon, Republic of Korea; 6 Kyungpook National University, School of Medicine, Department of Clinical Pathology, Daegu, Republic of Korea; 7 Kyungpook National University Hospital Culture Collection for Pathogens, Daegu, Republic of Korea

**Keywords:** Acinetobacter baumannii, multidrug resistance, blaOXA-66, blaOXA-23, F-plasmid, carbapenem

## Abstract

*Acinetobacter baumannii*, a strictly aerobic, non-fermentative, Gram-negative coccobacillary rod-shaped bacterium, is an opportunistic pathogen in humans. We recently isolated a multidrug-resistant *A. baumannii* strain KBN10P02143 from the pus sample drawn from a surgical patient in South Korea. We report the complete genome of this strain, which consists of 4,139,396 bp (G + C content, 39.08%) with 3,868 protein-coding genes, 73 tRNAs and six rRNA operons. Identification of the genes related to multidrug resistance from this genome and the discovery of a novel conjugative plasmid will increase our understanding of the pathogenicity associated with this species.

The members of the *Acinetobacter* group are non-fermentative, Gram-negative bacteria isolated from diverse environments, including human skin, soil, water and sewage. *Acinetobacter baumannii* and *A. junii* strains are opportunistic human pathogens with biomedical importance and are associated with nosocomial infections (van den Broek et al. 2009). *A. baumannii* holds particular interest in clinical microbiology because some strains of this species show resistance to almost all known antibiotics in clinical settings (Dijkshoorn et al. 2007).

We isolated a multidrug-resistant *A. baumannii* KBN10P02143 from the pus sample drawn from a surgical patient at the Kyungpook National University Hospital of South Korea in 2012. Antibiotic susceptibility testing with VITEK 2 showed that the strain is resistant to carbapenems, aminoglycoside antibiotics, extended cephalosporins, and folate pathway inhibitors, but susceptible to polymyxin antibiotics including colistin. To survey the genomic potential of extensively drug-resistant *A. baumannii*, we first cultured the KBN10P02143 strain at 37ºC on MacConkey agar. Then, genomic DNA was extracted using i-genomic BYF Mini Kit (iNtRON Biotechnology, Republic of Korea) following manufacturer’s protocol. Genome sequencing was performed using PacBio RS II single-molecule real-time (SMRT) sequencing technology (Pacific Biosciences, Menlo Park, CA, USA). A standard PacBio library with an average of 20-kb inserts were prepared and sequenced, yielding >142X average genome coverage. *De novo* assembly of the 61,433 reads with an average of 11,382 nucleotides (total, 699,233,841 bp) was conducted using the hierarchical genome-assembly process (HGAP) pipeline of SMRT Analysis v2.3.0 (Chin et al. 2013). Protein-coding genes were predicted by Prodigal v2.6.1. The signal peptides and transmembrane regions of the predicted genes were predicted using SignalP v4.1 and TMHMM v2.0, respectively. BLAST searches were performed against UniProt, Pfam, and COG databases to functionally annotate predicted genes. Ribosomal RNA, transfer RNA and miscellaneous features were predicted using RNAmmer v1.2, tRNAscan-SE v1.21 and Rfam v12.0. References for gene prediction and annotation can be found in Lee et al. (2015). Prophage regions were identified using PHAST web-based program (Zhou et al. 2011). Genome synteny of the plasmids was compared using Mauve v2.4.0 (Darling et al. 2004).

For phylogenetic analysis of *Acinetobacter* species, the nucleotide sequences of 16S rRNA genes and the amino acid (aa) sequences of proteins related to type IV secretion system (T4SS) in a plasmid were aligned using the MUSCLE alignment tool. Phylogenetic trees were reconstructed using the methods of neighbor-joining (NJ) with the Kimura-2-parameter model, maximum likelihood (ML) with the JTT model, and maximum parsimony (MP) in the MEGA6 package program (Tamura et al. 2013). Phylogenetic confidence was evaluated by the non-parametric bootstrap method with 500 replicates (Felsenstein 1985).

The genome consists of one circular chromosome (length, 4,086,879 bp) and one circular plasmid (52,517 bp) with 39.08% G+C content. Of the 3,981 genes encoded by this genome, 3,917 and 64 genes are present in the chromosome and plasmid, respectively. The coding regions that cover 3,605,312 bp (87.1% of the genome) encode 3,868 proteins (Table). Of them, 3,177 (79.8%) of the protein-coding genes were functionally assigned, while the rest of genes were annotated as hypothetical proteins. The chromosome harbors 18 rRNAs (six operons made up of 5S, 16S, and 23S, in that order) and 73 tRNA genes (Table). In addition, two intact prophage regions were detected in the chromosome. The prophage regions that include 133 coding DNA sequences are 97.3 kb (locus tags KBNAB1_0886 to KBNAB1_1021) and 94.9 kb (KBNAB1_1523 to KBNAB1_1659) long, respectively. Only half of the genes (42.86% and 42.11%) of the two prophage regions were homologous to those of the previously reported *A. baumannii* phage (GenBank accession number, NC_019541; Jeon et al. 2012), showing that prophage sequences can vary at the intra-species level of *A. baumannii*.

**TABLE:** General features of *Acinetobacter baumannii* KBN10P02143 complete genome

Attribute	Chromosome	Plasmid
Assembly size (bp)	4,086,879	52,517
Contigs	1	1
GC content (%)	39.11	36.42
DNA coding region (%)	87.10	86.86
Predicted ORFs	3,804	64
rRNA	18	0
tRNA	73	0
Genes assigned to COGs	2,710	53
Genes with Pfam domains	2,923	28
Genes with signal peptides	321	11
Genes with transmembrane helices	852	16

Similar to other *A.baumannii* strains, the KBN10P02143 genome exhibits genes encoding diverse virulence factors. For example, the presence of the *pgaABCD* gene cluster (KBNAB1_1395 to KBNAB1_1398) and *csu* operon (KBNAB1_1304 to KBNAB1_1309) indicates that this strain is capable of forming biofilms on abiotic surfaces. In addition, a gene cluster related to the biosynthesis of a siderophore (acinetobactin; KBNAB1_1131 to KBNAB1_1143) may play a role in successful colonisation of *A. baumannii* strains by obtaining iron from host tissues (Choi et al. 2009, Gaddy et al. 2012). The genome contains genes encoding diverse hemolysins (e.g., KBNAB1_0557) as well as the outer membrane protein A (*ompA*, KBNAB1_0575), both of which are necessary to induce potent inflammatory responses (Jun et al. 2013). These pathogenicity-related genes are found in the chromosome, but not in the plasmid.

Along with virulence, genomic information supports the multidrug resistance of KBN10P02143. For example, genes encoding seven beta-lactamases (Class A type, KBNAB1_2650 and KBNAB1_2518; Class D type, KBNAB1_1313 and KBNAB1_3206 homologous to OXA-23, and KBNAB1_2243 homologous to OXA-66; Class C type, KBNAB1_1159 and KBNAB1_3521) render the strain resistant to beta-lactam antibiotics (Walther-Rasmussen & Hoiby 2006). Of particular interest is class D *beta*-lactamases that play a crucial role in carbapenem resistance, with an active role of the AdeABC efflux system (KBNAB1_1972 to KBNAB1_1977; Lee et al. 2010). In addition, this genome encodes many antibiotic resistance genes that include aminoglycoside-modifying enzymes (aminoglycoside acetyltransferase, KBNAB1_1341), aminoglycoside phosphotransferase (KBNAB1_1338, KBNAB1_1851), streptomycin 3’-adenylyltransferase (KBNAB1_1343, KBNAB1_2641), dihydropteroate synthase (KBNAB1_2639 for sulfonamide resistance), dihydrofolate reductase (KBNAB1_3423), chloramphenicol acetyltransferase (KBNAB1_0725, KBNAB1_1342), macrolide 2’-phosphotransferase (KBNAB1_1352) and macrolide efflux protein (KBNAB1_1351). The presence of these genes shows that the strain would be capable of overcoming the stimuli induced by diverse antibiotics (Zhu et al. 2013). At least, it is obvious that the strain KBN10P02143 is resistant to carbapenems, aminoglycoside antibiotics and folate pathway inhibitors since the resistance to the three antibiotics is supported by both genomic and experimental data (see the VITEK 2 result above).

The plasmid pKBN10P02143 is putatively categorised into F-plasmid because 17 of the 64 protein-coding genes encode the conjugative apparatus and T4SS. Although the replicase of pKBN10P02143 cannot be classified by the present replicon typing method, the enzyme contains the PF03090 domain that is grouped to the GR6 type of replication (Bertini et al. 2010). This replicase is more similar to that of *A. junii* NIPH 182 (97% identity at the aa level) than those of other *A. baumannii* isolates (e.g., <39%, compared to the strain 1656-2). Since all the phylogenetic trees of 16S rRNA sequences reconstructed using NJ, ML and MP support that *A. junii* is evolutionarily distant from *A. baumannii* (Fig. 2A), lack of sequence homology between KBN10P02143 and other *A. baumannii* strains supports the possibility that the F-plasmid of KBN10P02143 would be horizontally transferred rather than vertically inherited. This inference is also supported by genome synteny analysis. We conducted whole genome sequence alignment of the pKBN10P02143 with the sequences of seven F-plasmids from other *A. baumannii* strains (Liu et al. 2014) by using Mauve software. We found that many regions of the pKBN10P02143 F-plasmid are absent in the F-plasmids of other *A. baumannii* strains, although the T4SS region is conserved across all isolates (Fig. 1). To investigate the abnormal evolutionary affinity of T4SS of pKBN10P02143 to the plasmids of *A. junii* and *A. beijerinckii*, the aa sequences of proteins coded by *traB*, *traC*, *traD* and *traG*, the major gene components of T4SS, were phylogenetically analysed (Fig. 2B-E). All the phylogenetic trees reconstructed using NJ, ML and MP algorithms support that the genes of pKBN10P02143 are grouped together with those of *A. junii* and *A. beijerinckii*, and that they are evolutionarily distant from those of other *A. baumannii* strains. The phylogenetic results along with sequence similarity comparison indicate that the F-plasmids are horizontally exchanged between two different species, rather than being vertically inherited from the most recent common ancestor. Consequently, diverse genetic information, including that associated with virulence and multidrug resistance, can be non-vertically transferred from/to other pathogenic/non-pathogenic *Acinetobacter* spp., as exemplified by the F-plasmid.

**Fig. 2:**
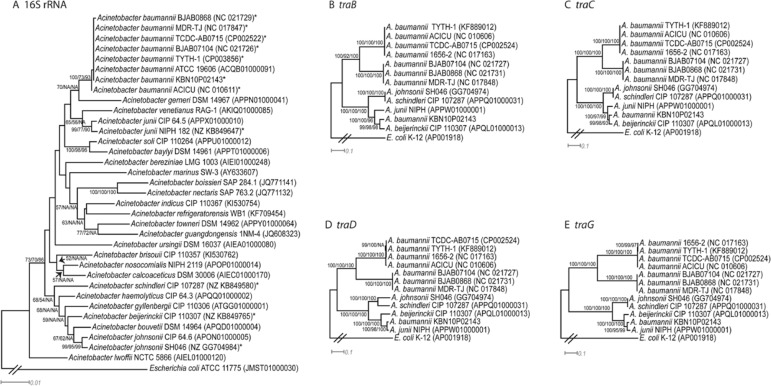
: phylogenetic trees of *Acinetobacter* spp. by using (A) 16S rRNA gene; (B) *traB*; (C) *traC*; (D) *traD*; and (E) *traG*. The DNA and amino acid (aa) sequences were aligned using MUSCLE. Tree reconstruction was performed using the method of neighbor-joining (NJ), maximum likelihood (ML) and maximum parsimony (MP). The scale bars indicate the units of the number of DNA or aa substitutions per site that is calibrated by the Poisson correction method. All positions containing gaps and missing data were eliminated. Numbers adjacent to nodes are bootstrap support values of NJ, ML and MP in that order. ‘NA’ indicates the bootstrap support values lower than 50%. Taxa used for phylogenetic analysis of *tra* genes were indicated by asterisks in the phylogenetic tree of 16S rRNA gene.

**Fig. 1:**
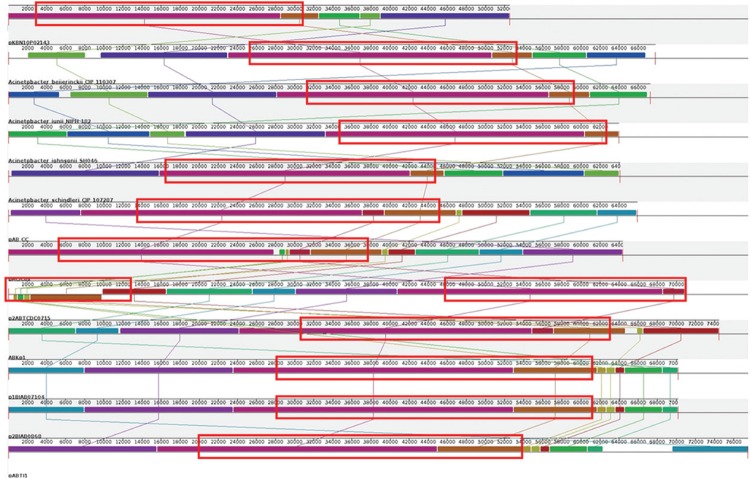
: multiple whole sequence alignments of the plasmids pKBN10P02143, pAB_CC (*Acinetobacter baumannii* TYTH-1, KF889012), pACICU2 (*A. baumannii* ACICU, NC_010606), p2ABTCDC0715 (*A. baumannii* TCDC-AB0715, CP002524), ABKp1 (*A. baumannii* 1656-2, NC_017163), p1BJAB01704 (*A. baumannii* BJAB07104, NC_021727), p2BJAB0868 (*A. baumannii* BJAB0868, NC_021731), pABTJ1 (*A. baumannii* MDR-TJ, NC_017848), contigs acLZq-supercont1.5.C13 (*A. beijerinckii* CIP 110307, APQL01000013), supercont1.11 (*A. johnsonii* SH046, GG704974), acLrq-supercont1.1.C1 (*A. junii* NIPH, APPW01000001), and acLsx-supercont1.16.C31 (*A. schindleri* CIP 107287, APPQ01000031) using the Mauve tool. The T4SS regions are indicated in redlined boxes.

The complete genome sequence of *A. baumannii* strain KBN10P02143 has been deposited at DDBJ/EMBL/GenBank under the accession numbers CP013924 (chromosome) and CP013925 (plasmid). The strain is available from Kyungpook National University Hospital Culture Collection for Pathogens.
